# Feasibility of Pressure-Retarded Osmosis for Electricity Generation at Low Temperatures

**DOI:** 10.3390/membranes11080556

**Published:** 2021-07-23

**Authors:** Elham Abbasi-Garravand, Catherine N. Mulligan

**Affiliations:** Department of Building, Civil and Environmental Engineering, Concordia University, 1455 de Maisonneuve Blvd. W., Montreal, QC H3G 1M8, Canada; sanaz.abbasi@Concordia.ca

**Keywords:** osmotic power, salinity gradient energy, pressure-retarded osmosis, PRO membrane, low temperature

## Abstract

A membrane-based technique for production of pressure-retarded osmosis (PRO) is salinity gradient energy. This sustainable energy is formed by combining salt and fresh waters. The membrane of the PRO process has a significant effect on controlling the salinity gradient energy or osmotic energy generation. Membrane fouling and operating conditions such as temperature have an extreme influence on the efficiency of the PRO processes because of their roles in salt and water transportation through the PRO membranes. In this study, the temperature impact on the power density and the fouling of two industrial semi-permeable membranes in the PRO system was investigated using river and synthetic sea water. Based on the findings, the power densities were 17.1 and 14.2 W/m^2^ at 5 °C for flat sheet and hollow fiber membranes, respectively. This is the first time that research indicates that power density at low temperature is feasible for generating electricity using PRO processes. These results can be promising for regions with high PRO potential that experience low temperatures most of the year.

## 1. Introduction

Osmotic power, also known as salinity gradient energy, has recently received a lot of interest as a sustainable energy source due to greenhouse gas emissions and resource depletion. For the generation of electric power based on salinity gradient energy, many technologies such as pressure-retarded osmosis (PRO), reversed electrodialysis (RED), electrical double-layer capacitor (EDLC), and power generation via vapor pressure difference (VPD) are being developed. Among these, intensive technological developments have been done on PRO [1]. Pressure-Retarded Osmosis (PRO) has drawn remarkable recognition recently [2,3,4,5]. In PRO technology, salinity/gradient is used as a driving force to produce osmotic power [6,7,8,9]. A difference in osmotic pressure between the two sides of the membrane (∆π) causes this and as a result, water with less salinity from the feed solution is driven to a pressurized draw solution with higher salinity [10]. This energy can be harvested wherever surface/clean water from rivers encounters salt water from a sea, bay or saline lake [8]. Industrial effluents and brines from desalination plants [11] are other potential sources of high salinity water. The overall capacity of the osmotic energy in the world has been estimated to surpass 2 TW [12]. A back pressure is applied on the draw solution and electric energy is produced by depressurizing the pressurized draw solution through a turbine [13]. Compared to other osmotically driven membranes such as forward osmosis (FO) and reverse osmosis (RO), in PRO, water flows through the membrane from the fresh side to the concentrated salt side, which is under hydraulic pressure (∆π > ∆P). However, hydraulic pressure (∆P) is nearly zero and water passes through the membrane from the fresh side to the concentrated salt side in FO, and in RO water passes through the membrane from the concentrated salt side to the fresh side by applying hydraulic pressure (∆P > ∆π) [1].

The semi-permeable membrane of the PRO process has a significant effect on controlling the salinity gradient energy or osmotic energy production [14]. Like other membrane processes, operating factors such as temperature, pressure, concentration of the solution and flow rates have a major effect on PRO process efficiency due to their roles in transportation of salt and water through the PRO membranes [4].

Although much research has been done on PRO, there is very limited research on the impact of temperature on the efficiency of the PRO processes [15,16,17]. As temperature affects membrane permeability, fouling propensity, reverse salt diffusion, and structural parameters and, consequently, it also influences the performance of the PRO process and osmotic power generation [16].

As is well known, temperature varies seasonally depending on the geographical locations. Regions in the world with high osmotic power potential are in cold regions such as many off-grid communities in Canada and Norway [18,19]. PRO can be considered to be an option for replacement of diesel generators in these off-grid communities to minimize the environmental effects due to combustion [20]. Therefore, it is very important to determine the impact of low temperature on the efficiency of the PRO process and power generation to design them under these conditions. Temperature can affect the structure and permeability of the membrane and reverse salt diffusion [21]. 

The aim of this study is to understand the role of low temperature on the efficiency of the PRO process to enable implementation in northern regions. In this research, semi-permeable membranes were used to investigate the influence of temperature on the PRO process at high flow rates and pressures.

## 2. Materials and Methods

### 2.1. Chemicals

Sodium chloride and calcium chloride in a ratio of 26 (wt./wt.) were used to prepare synthetic sea water. The reason for adding calcium chloride was to determine the impact of calcium ion on fouling of membrane in PRO processes. The ratio of 26 was chosen according to the ratio of Na/Ca in sea water [22,23]. In this research, the concentration of the salt water was 30 g/L to simulate the salinity to that of the Saint Lawrence River at its estuary [24]. Sodium chloride (NaCl-10 kg-S271-10) as a reagent salt and calcium chloride dihydrate (${\mathrm{CaCl}}_{2}$-3 kg-C79-3) as an additive to salt water were obtained from Fisher Scientific Co. to make synthetic sea water in this study. All experiments were done at Concordia University using a PRO experimental set up provided by the Hydro-Quebec Research Institute (Shawinigan, QC, Canada).

### 2.2. Water Quality

Four river water samples were collected from the Saint Lawrence River at Montreal in the winter. To examine physiochemical characteristics of the river water, parameters such as iron, total organic carbon (TOC), silica, alkalinity, hardness, pH, salinity, conductivity, turbidity, sodium, iron, calcium, magnesium, and potassium were measured. Among these parameters, turbidity, hardness, monovalent and divalent cations, silica, and total organic carbon (TOC) play an essential role in membrane fouling. The analytical methods for measurement of these parameters are detailed in previous publications [6,12,25]. Table 1 summarizes the average physiochemical characteristics of the river water samples determined in the laboratory.

### 2.3. PRO Unit

#### 2.3.1. Membrane and Spacer

##### Flat Sheet Membrane

**Figure 1:**
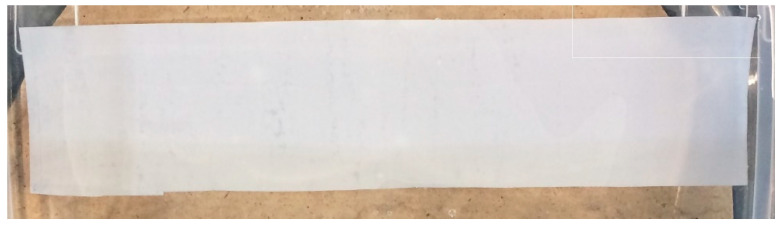
Flat Sheet Membrane.

A thin-film composite (TFC) membrane with a hydrophilic support layer was purchased from Porifera Inc. and was used as the PRO membrane in this research [26]. The active surface area of the membrane was 0.00875 m^2^. Specific parameters of the used TFC membrane, the procedure for using and installing the membrane and spacers were the same as in previous work [6,12,25]. Figure 1 shows the flat sheet membrane.

##### Hollow Fiber Membrane

**Figure 2:**
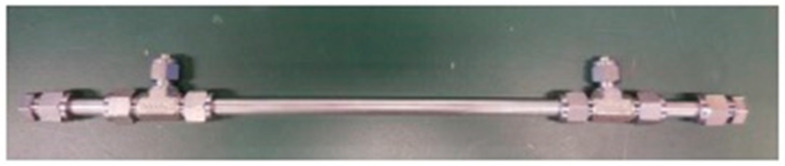
Hollow Fiber Membrane Module.

The used hollow fiber membrane was a cellulose triacetate (CTA) one provided by a company in Japan (according to a signed non-disclosure agreement (NDA), the name of the company cannot be disclosed). The active surface area of the membrane was 0.057 m^2^. Key parameters of the used membrane cannot be published due to confidentiality agreements. Figure 2 shows the hollow fiber membrane module.

#### 2.3.2. PRO Experiments

**Figure 3:**
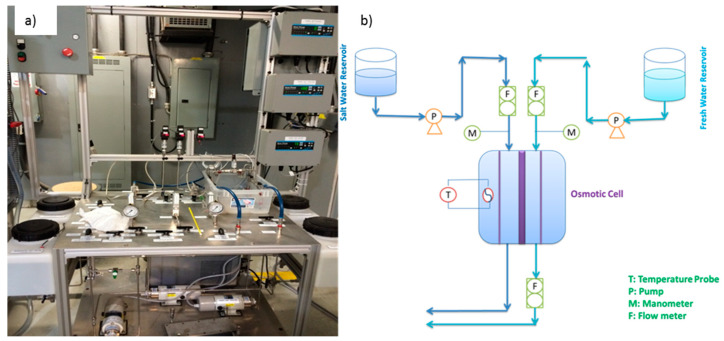
(**a**) PRO membrane setup at Hydro-Quebec Research Institute, (**b**) schematic of PRO membrane setup [6,12,25].

The PRO membrane setup was the same as previous work [6,12,25]. Figure 3a shows this provided setup by Hydro-Quebec. The materials and devices that were used for the PRO membrane test system were similar to a previous study except for the pumps and flowmeters. The previous pumps were replaced by new ones to run the system at greater flow rates and pressures. The pumps were purchased from Cole-Parmer Co. The maximum capacity of the pumps for pressure and flowrate were 1100 psi (7584.2 kPa) and 188 mL/min, respectively. Figure 3b is a sketch of the experimental setup and shows different parts of the apparatus such as fresh and saltwater reservoirs, pumps, flowmeters, water bath, temperature probe, and the osmotic cell.

**Figure 4:**
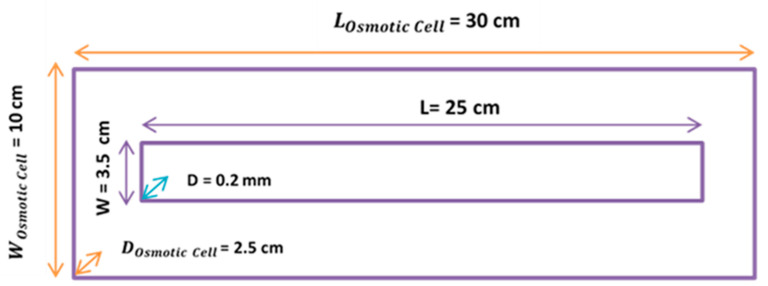
Osmotic cell dimensions [6,25].

Figure 4 demonstrates the dimensions of the used osmotic cell. Flowmeters (model: Masterflex) were purchased from Cole-Parmer Co. with flow rates in the range of 0–200 mL/min. These flowmeters were used to measure the inlet and outlet flow rates of the fresh water during the PRO experiments. The permeate flow rate was the difference between these inlet and outlet flow rates.

Equation (1) was used to calculate the permeate flux [2,6,25]:

(1) Flux (Lm2·h)=[Permeate flow rate (mLmin)Cartridge area (m2)]×0.06

0.06 represents the unit conversion for mL/min to L/h.

Equation (2) was used to calculate power density [8,27].

(2) $$W={\;J}_{w}\cdot \Delta P$$

W: Power density (W/m^2^)
$J_{w}$: PRO membrane permeate flux ($\frac{m^{3}}{m^{2}\cdot s}$)
ΔP: Differential hydraulic pressure across the membrane (Pa)

The flow rates on both salt and fresh sides were 106 mL/min. A range of hydraulic pressures from 0.48 to 15.4 (48 to 1540 kPa) were applied on the draw solution in recent PRO research [7,28,29,30,31,32,33]. In this study, the applied hydraulic pressure on the draw side was 6 bars (600 kPa) due to the device limitation. Different temperatures (5, 15, 25, and 35 °C) were used in this study. These were chosen at water temperature normally varies in numerous climates between 12 to 35 °C [34] and a lower temperature (5 °C) was added to evaluate application for northern climates. A water bath was used to keep the temperature constant, and a thermometer was also placed in the water bath to control the accuracy of the temperature during the tests. The accuracy of the measurement of the temperatures was ±0.5 °C. For the fouling trials, the initial conditions such as pressure, temperature, and salt concentrations were sustained and were 600 kPa (6 bars), 5 °C, and 30 g/L, respectively. The pressure drops were negligible at the input and output of the fresh and salt sides in the PRO setup. All experiments were carried out the same way as outlined in the previous work [6,12,25]. During the experiments, inlet and outlet flowrates, temperature, and pressures were continuously measured and recorded in an interval of 60 s.

## 3. Results and Discussion

### 3.1. Investigation of Temperature Effect on Permeate Flux

In this experiment four different temperatures (5, 15, 25, and 35 °C) were used to explore the influence of temperature on permeate flux for two commercial semi-permeable membranes with different configurations (flat sheet and hollow fiber). These temperatures were selected to reflect the seasonal variation in many climates. The minimum operating temperature that the membranes could tolerate was 5 °C based on the data sheets provided by the companies. Therefore, the temperature of 5 °C was selected to evaluate the performance of the PRO processes at low temperature in this study. The applied pressure, salt concentration on draw side, flow rates on the feed and draw sides were 600 kPa (6 bars), 30 g/L, and 106 mL/min, respectively. 

**Figure 5:**
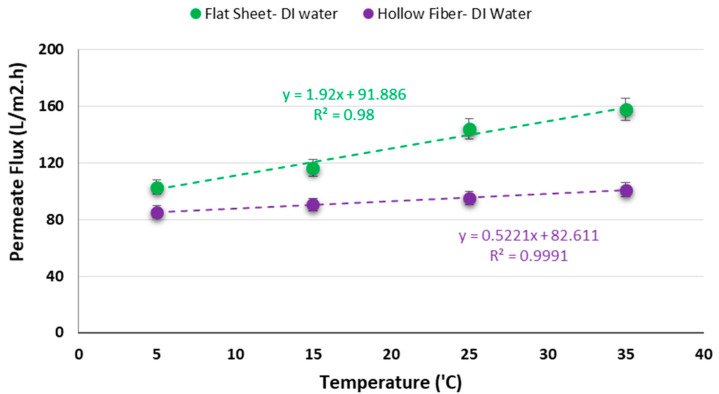
Impact of temperature on permeate flux for two different membrane modules.

In the light of the results shown in Figure 5, by increasing the temperature, the permeate flux increased which as well is in accordance with the results published by Heo et al. [15]. When the temperature increases, the feed water viscosity reduces and mass transfer becomes greater and as a result, the flux increases [14,35]. The rise in the temperature, on the other hand, can increase the driving force (the variation of the osmotic pressure between two sides of the membrane) and as a result, enhances the water flux and power density and improves the performance of the PRO processes [17]. The maximum permeate fluxes were 101.1 and 157.7 L/m^2^h at 35 °C for hollow fiber and flat sheet configurations, respectively. The minimum permeate fluxes were 85.3 and 102.9 L/m^2^h at 5 °C, for hollow fiber and flat sheet configurations, respectively.

The power densities were calculated using Equation (2) for both membrane configurations. The maximum and minimum power densities were 26.3 and 17.1 W/m^2^ for the flat sheet configuration and 16.8 and 14.2 W/m^2^ for the hollow fiber configuration at 35 and 5 °C, respectively. As expected, the power density decreased by reducing the temperature regardless of the membrane configuration. As mentioned above, the osmotic pressure and diffusivity decreases by reducing the temperature, while the viscosity of the feed solution increases, and this leads to a decrease in the mass transfer and ultimately reduces the water permeate flux and power density. The result shows that the lowest amount of power density occurred at 5 °C for the two of them (flat sheet and hollow fiber modules) and it is still higher than the minimum required power density (3–5 W/m^2^) to generate economic PRO power [8,32,36,37,38,39]. This may be associated with the impact of the temperature on reverse salt flux. Although the water permeate flux reduces by decreasing the temperature, it has been proven that the salt diffusion from the draw side to the feed side also decreases and consequently, internal concentration polarization (ICP) reduces, which contributes to an improvement in the PRO performance [27,40,41]. This behavior can be linked to the membrane structure and effect of the temperature on it. When the temperature decreases, the membrane pores tend to shrink and correspondingly the salt diffusion from draw side to the feed side decreases and this leads to an improvement of PRO performance. The results in this research shows that the influence of the temperature on the hollow fiber membranes is less compared to the flat sheet membranes. The reason can be related to the structure and materials of both membranes and their reactions to the temperature. Further investigation is needed to understand why the temperature influences the hollow fiber membrane less compared to the flat sheet membrane.

Moreover, Table 2 and Table 3 indicate a comparison between the value of power densities in this study and other reported studies in the literature for both commercial flat sheet and hollow fiber membranes. According to this comparison, the amount of power density at 5 °C in this study is not only greater than the amount of that for generating economic PRO power but also it is greater than that in other available commercial membranes when their temperatures were equal or above 20 °C. Except for the last case in Table 3 that reports a power density of 17.1 W/m^2^ which was achieved at significantly higher operating conditions compared to this study (3000 kPa (5 times more), 40 °C (8 times more) and 60 g/L (2 times more)). The results obtained in this study are promising for regions with high PRO potential that experience low average temperatures most of the year.

### 3.2. Salt Concentration Effect on Permeate Flux at Low Temperature

In this experiment, a draw solution with three different salinities was used to research the impact of salinity on permeate flux and consequently the performance of PRO system at low temperature. The used salt concentrations were 30, 60 and 90 g/L (0.5, 1, and 1.5 M). The temperature was fixed at 5 °C for both membrane modules. Initial conditions were constant and were the same as other experiments previously mentioned.

**Figure 6:**
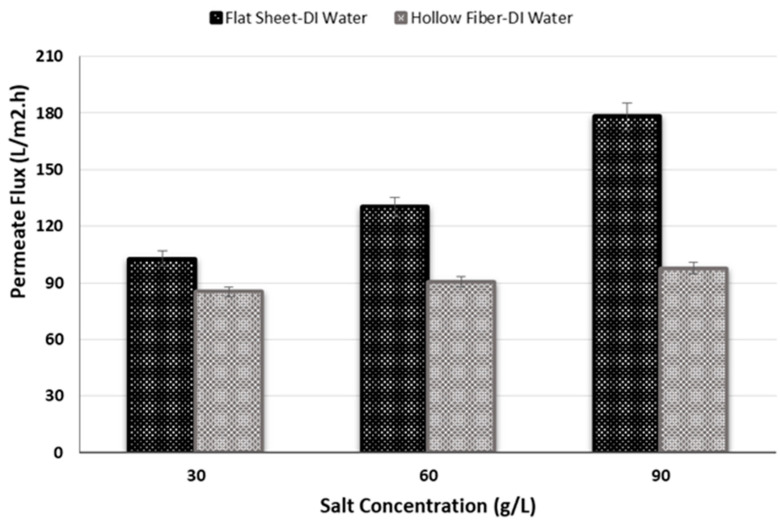
Influence of salt concentration on permeate flux for two different membrane modules.

For both membrane modules, while a positive association was found within the salt concentration on the draw side and the permeate flux it was particularly significant for the flat sheet membrane (Figure 6). In other words, the permeate flux augments as well as the concentration of salt on the draw side increases. This occurs due to the osmotic driving force. When the salt concentration increases, the osmotic driving force increases as well in addition to the permeate flux. This pattern has also been noted by Duong et al. [49]. The maximum permeate fluxes occurred at 90 g/L and were 97.8 and 178.3 L/m^2^h for hollow fiber and flat sheet membranes, respectively. Further investigation is required to determine the critical values. 

### 3.3. Influence of Pressure on Permeate Flux and Power Density at Low Temperature

**Figure 7:**
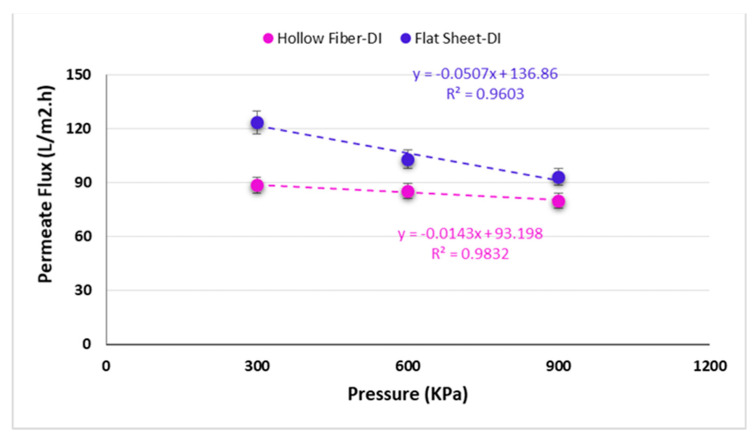
Influence of pressure on permeate flux for two various membrane modules.

In this trial, a pressure in a range of 0 to 900 kPa was applied on the draw side to compare the effect of pressure on permeate fluxes and power densities for two commercial semi-permeable membranes in PRO mode. As mentioned above, initial conditions such as feed and draw flow rates, salt concentrations, and temperature were the same for both membranes. All tests were conducted at 5 °C. The other conditions such as salt concentration and feed and draw side flow rates were fixed at 30 g/L and 106 mL/min throughout the trial, respectively. The quantity of water that can flow through the membrane lessens by increasing the pressure and accordingly the permeate flux [50,51,52]. These results are in line with the observations made by She et al. [28]. As the results show, by increasing the pressure, the permeate fluxes decline while the power densities increase (Figure 7).

**Figure 8:**
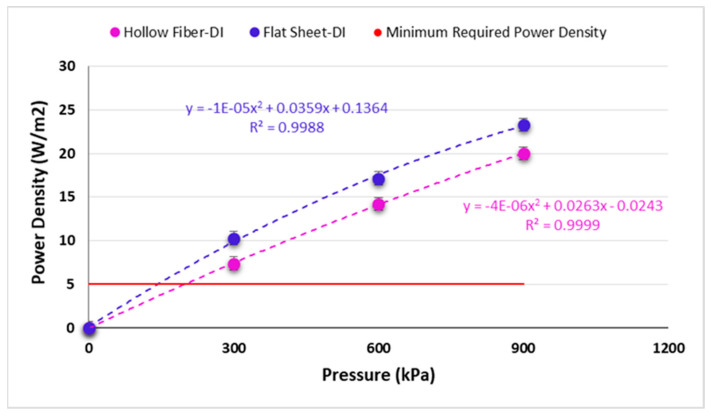
Power density results using two different membrane modules.

Based on the results indicated in Figure 8, upon increasing the applied pressure, the power density also rises to 20 and 23 W/m^2^ at 900 kPa (9 bars) for hollow fiber and flat sheet membranes, respectively. A 2nd order polynomic trend in Figure 8 is shown indicating the nonlinear relationship between pressure and power density. This has also been shown by the exponential model and experimental results by Touati et al. [21]. They also showed that as the pressure is increased eventually a maximum is obtained when theoretically applied pressure is half the osmotic pressure difference. However, it is usually less than that due to the ICP effect from the increased feed concentration. 

As is well known, the PRO membrane must have a power density higher than 3–5 W/m^2^ to be economical for PRO power production [8,32,36,37]. The maximum power densities recorded in this research were reached at 20 and 23 W/m^2^ when the flow rate and pressure were 106 mL/min and 900 kPa (9 bar) respectively. It has also been indicated by Cath et al. [53] that PRO membranes should have higher water fluxes than 50 L.m^2^-h at pressures higher than 800 psi as was found in this study. These results show the feasibility of the PRO technology at low temperature. 

### 3.4. Impact of Fouling on Permeate Flux at Low Temperature

In this test, the influence of fouling on two different membrane configurations in the PRO mode was studied. Entire fouling experiments were conducted at a fixed temperature (5 °C), salinity (30 g/L) and pressure (600 kPa). The used draw solution was a synthetic sea water while the feed water was river water. The tests were run for 180 min. The flow rates for all trials on two sides (feed and draw) were 106 mL/min. All tests were carried out in triplicate. The reason for running the tests for 180 min was due to the equipment limitation for reservoirs. Fouling kinetics in the PRO system have been investigated in a previous study [25]. Recirculation of the draw solution did not occur in the system because the PRO experimental setup was an open system and was continuously drained into a waste disposal system. This indicates that the draw solution was not diluted and consequently, the salt concentration remained the same during the tests. Hence, in the trials, dilution had no impact on the fouling and the flux decrease.

**Figure 9:**
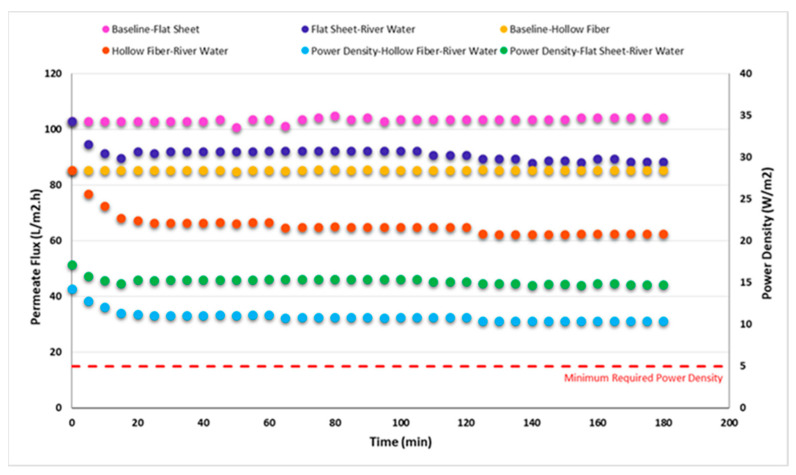
Impact of fouling on permeate flux and power density for two diverse membrane modules.

As Figure 9 shows, against the flat sheet membrane, fouling occurred faster in the hollow fiber membrane. This result is in accordance with the findings achieved by Bodík et al. [52]. Permeate fluxes decreased by 12.9% and 20% in the flat sheet and hollow fiber membranes, after passing 15 min, respectively. As Figure 9 demonstrates, in the fouling experiments, permeate fluxes decreased at the beginning and then became nearly constant. Lee et al. [52] also reported the same pattern regarding permeate flux shifts over time. A potential advantage of running at low temperatures, is that salt accumulation at the membrane would be less than at higher temperatures which could decrease membrane fouling [21].

For the two different membranes, the power density was determined as the product of the permeate flux multiplication in applied hydraulic pressure (Equation (2)) to compare the impact of the fouling on power density. Based on the results indicated in Figure 9, the power density reduced from 17.1 to 14.7 W/m^2^ (14% decrease over 180 min) using the flat sheet membrane while it declined from 14.2 to 10.4 W/m^2^ (27% decrease over 180 min) using the hollow fiber membrane, respectively. The results from this test shows that although the fouling reduces the power density in PRO membranes, the generated power densities are still much higher than the minimum required power density (5 W/m^2^). This means that salinity gradient energy can be feasible in cold regions. According to the findings in the previous work [6], inorganic and organic foulants such as iron, aluminum, calcium, sodium, silica, humic substances, polysaccharides and proteins have a significant role in causing the fouling on the flat sheet membranes when synthetic sea water and river water were used as draw solution and feed water, respectively. However, further investigation is needed to identify the main foulants and the fouling mechanisms in the hollow fiber membranes as well as to understand why the fouling rate in the hollow fiber membrane was more than that of the flat sheet membrane when the experimental conditions were the same for both membranes. If pretreatments are needed to reduce fouling, their energy must be minimized to allow an overall economically feasible energy production process. Other approaches are to develop membranes that are resistant to fouling but are pressure tolerant, and highly water permeable with a high solute rejection (greater than 99%) [53].

Chia et al. [54] discussed some potential environmental impacts of PRO such as noise, potential risk of contaminants entering water bodies and the requirements for large amounts of water. Closed loop or industrial effluents of high salinity as the draw solution could reduce water needs. PRO, however, is a clean energy and it was estimated by Touati and Tadeo [55] that global GHG emissions could be reduced by 2741 megatons of GHC by 2030 by PRO implementation. Minimal environmental impacts at estuaries are expected. The impact of construction of PRO facilities would be similar to any other facility such as desalination [56]. 

## 4. Conclusions

In this study, the influence of low temperature on the performance of the PRO processes was examined using two different commercial semi-permeable membranes. According to the results, a positive relation was obtained between the temperature and the permeate flux for both hollow fiber and the flat sheet membranes. In other words, by raising the temperature, the permeate flux increased as well. It was interesting to observe that although the permeate flux and produced power density declined by decreasing the temperature, the amount of produced power density at the lowest temperature used in this research (5 °C) was still higher than the minimum required power density. In addition, the achieved results indicated that the salt concentration of the draw solution had an immediate impact on the permeate flux and produced power density in PRO mode at low temperature. Results demonstrate that the permeate flux was adversely affected by the applied pressure. The permeate flux reduced upon rising the applied pressure on the draw solution and therefore the obtained power density increased when the temperature was low for both the hollow fiber and the flat sheet membranes in PRO mode. In addition, the influence of the fouling on the efficiency of both membrane modules, was examined using river water and synthetic sea water as feed water and draw solutions, respectively. The fouling results indicated that the permeate flux decreased by time for both flat sheet and the hollow fiber membranes. However, the fouling effect on the hollow fiber membrane was more severe than that on the flat sheet membrane. Based on the fouling results, the produced power density decreased over time, but the amount of the power density after three hours was still much higher compared to the minimum required power density in PRO processes for both membrane modules at low temperature. Therefore, this research shows that the pressure-retarded osmosis can be feasible at low temperature in cold regions as a sustainable energy source. As physiochemical characteristics of seawater under real conditions differ from the artificial sea water characteristics, further investigations are required to understand the key controlling factors influencing both fouling and performance of the PRO processes at low temperatures for various membrane configurations. Moreover, evaluation of the performance and efficiency of the PRO systems in cold regions at pilot scale using larger membrane modules is needed to understand the limitations of the practical application of this technology. Furthermore, additional research is needed to identify the economic feasibility and environmental aspects of the PRO system on the ecosystem over the long term.

## Figures and Tables

**Table 1:** Physiochemical characteristics of the river water.

Parameters	River Water
Total iron (mg/L Fe)	0.16 ± 0.01
TOC (mg/LC)	8.21 ± 0.4
Silica (mg/LSiO_2_)	1.38 ± 0.07
Suspended solids (mg/L)	1.00 ± 0.05
Total alkalinity (mg/L as ${\mathrm{CaCO}}_{3}$)	6.00 ± 0.25
Hardness (mg/L as ${\mathrm{CaCO}}_{3}$)	6.00 ± 0.25
pH	7.49 ± 0.38
Salinity (%)	0.01 ± 0.001
Conductivity (μS/cm)	21.87 ± 1.09
Turbidity (NTU)	2.34 ± 0.12
Dissolved solids (mg/L)	10.19 ± 0.51
Sodium (mg/L)	3.29 ± 0.17
Calcium (mg/L)	6.61 ± 0.33
Magnesium (mg/L)	1.70 ± 0.1
Potassium (mg/L)	0.74 ± 0.04

**Table 2:** Comparison between PRO experimental performances in this study and other reported studies in the literature using commercial flat sheet membranes.

Temperature (°C)	Membrane	Salinity Draw Side (g/L)	Feed Solution	Draw Solution	Pressure (kPa)	Power Density (W/m^2^)	Ref.
5	TFC-Flat Sheet	30	River Water	Synthetic Sea Water	600	17.1	This Study
25	TFC-Flat Sheet	30	River Water	Synthetic Sea Water	600	24	This Study
25	HTI-Flat Sheet	30	Synthetic Municipal Waste Water	Salt Water	900	1.2	[28]
25	HTI-CTA-Flat Sheet	35	Deionized Water	Salt Water	970	2.7	[7]
20	HTI-CTA-Flat Sheet	30	River Water	Synthetic Sea Water	800	1.51	[30]
22	HTI-CTA-Flat Sheet	30	Deionized Water	Sea Water	400	0.57	[42]
25	Toray Chemical Korea-Flat Sheet	35	Tap Water	Salt Water	1040	1.8	[43]
20	GKSS Germany-TFC-Flat Sheet	30	Fresh Water	Sea Water	1100	2.7	[44]
20	Osmonics Inc. USA-CA-Flat Sheet	23.5	Fresh Water	Sea Water	820	1.6	[44]

**Table 3:** Comparison between PRO performance in this study and other reported studies in the literature using commercial hollow fiber membranes.

Temperature (°C)	Membrane	Salinity (g/L)	Feed Solution	Draw Solution	Pressure (kPa)	Power Density (W/m^2^)	Ref.
5	CTA-Hollow Fiber	30	River Water	Synthetic Sea Water	600	14.2	This Study
25	CTA-Hollow Fiber	30	River Water	Synthetic Sea Water	600	15.9	This Study
25	Toyobo-CTA-Hollow Fiber	22	Permeate Water from Desalination Plant	Sea Water	1500	3.1	[45]
40	Toyobo-CTA-Hollow Fiber-10 inch	60	Waste Water	Brine from SWRO	2500	13.5	[46]
-	Toyobo-Hollow Fiber-10 inch	60	Treated Sewage	Concentrated Brine from SWRO System	2500	7.7	[47]
-	Toyobo-Hollow Fiber-10 inch	60	Treated Sewage	Concentrated Brine from SWRO System	2900	4.4	[47]
40	Toyobo-Hollow Fiber-10 inch	60	Treated Waste Water	SWRO Brine	3000	13.3	[48]
40	Toyobo-Hollow Fiber-5 inch	60	Treated Waste Water	SWRO Brine	3000	17.1	[48]

## Data Availability

Not applicable.
